# Effect of Pt Loading Methods of Pt/CeO_2_ Catalysts Derived from Ce-BTC as Support on Catalytic Oxidation of Toluene

**DOI:** 10.3390/molecules31142424

**Published:** 2026-07-10

**Authors:** Xinxin Jin, Zhihao Zhao, Panpan Tian, Zhen Song, Zhiping Zhang, Yujun Zhu

**Affiliations:** 1 Key Laboratory of Functional Inorganic Material Chemistry, Ministry of Education, School of Chemistry and Materials, Heilongjiang University, Harbin 150080, China; au.zn.zn@163.com (X.J.); zhangzhiping@hrbust.edu.cn (Z.Z.); 2 School of Materials Science and Chemical Engineering, Harbin University of Science and Technology, Harbin 150040, China; z18845761449@163.com (Z.Z.); tianpanpan0@163.com (P.T.); 18875704925@163.com (Z.S.); 3 School of Pharmacy and Health Sciences, Shaoxing Institute of Technology, Shaoxing 312000, China

**Keywords:** Pt loading, Ce-BTC MOF precursor, CeO_2_ as support, catalytic oxidation

## Abstract

Three Pt/CeO_2_ catalysts were prepared via thermal reduction with different Pt introduction sequences: impregnation (IM), reverse impregnation (RIM), and reduction–co-assembly (RCM). Their physicochemical properties and catalytic oxidation activity for toluene were compared over IM-prepared Pt_pre_/CeO_2_, RIM-prepared Pt/CeO_2pre_, and RCM-prepared Pt_pre_/CeO_2pre_. Different Pt loading methods affect the distribution, particle size, chemical state of Pt, and the metal–support interaction on the catalyst surface. Among them, the Pt_pre_/CeO_2pre_ catalyst exhibits a high oxygen vacancy concentration and the best low-temperature reduction characteristics. Its high Ce^3+^/Ce^4+^ ratio, lattice oxygen content, and the content of Pt with active valence states (Pt^0^, Pt^2+^) are all conducive to the catalytic oxidation of toluene. The content of different valence states of Pt, dispersion degree, and cluster size of Pt on the catalyst, as well as oxygen vacancies and the valence state of Ce, were evaluated by XPS, Raman, H_2_-TPR, in situ infrared, TEM, and N_2_ adsorption–desorption tests, clarifying the reasons for the high activity of the catalyst. This provides an experimental basis and contributes strategies for the development of noble metal-loaded oxide catalysts prepared from metal–organic frameworks as precursors.

## 1. Introduction

Volatile Organic Compounds (VOCs) are organic pollutants characterized by high vapor pressure (>133.32 Pa) at room temperature and boiling points ranging from 50 to 260 °C. These compounds exhibit properties such as high volatility, low water solubility, and strong reactivity, typically containing functional groups like carbon–hydrogen (C-H) and carbon–oxygen (C-O) bonds in their molecular structures [1,2]. Based on differences in chemical composition, VOCs can be categorized into four major groups: alkanes, aromatic hydrocarbons, oxygenated hydrocarbons, and halogenated hydrocarbons. Among these, aromatic hydrocarbons, particularly the benzene series (benzene, toluene, xylene), demonstrate significant toxicity. Toluene is listed as a priority control pollutant due to its extended half-life of up to 7 days and a bio-degradation rate of less than 10% [3]. Effectively balancing the relationship between the economic cost of catalysts and the degradation efficiency of toluene remains a prominent research focus in chemical engineering and thermal catalysis. Catalytic oxidation technology, also termed thermal catalysis, has emerged as a core technique for VOC degradation owing to its low operational costs and high catalytic efficiency [4]. Nevertheless, the rational design of low-temperature, high-activity catalysts continues to pose significant challenges. Current research hotspots in thermal catalysts primarily focus on three aspects: efficient loading of noble metals, synergistic of physicochemical properties in transition metal oxide catalysts, and the design of catalyst supports with tailorable high-porosity structures.

Metal–organic frameworks (MOFs) serve as ideal precursors or supports for designing catalytic materials and enhancing their performance, owing to their highly ordered porous structures, tunable pore sizes, and chemical modifiability [5,6]. Firstly, catalysts derived from MOF precursors possess high specific surface areas and uniformly distributed active sites. The specific surface area of MOFs can exceed 2000 m^2^/g (e.g., UiO-66, ZIF-8), significantly surpassing that of traditional metal oxides (e.g., ~300 m^2^/g for SiO_2_), thereby providing ample interfaces for reactant adsorption and diffusion [7]. Regarding thermal catalysts, CeO_2_ has attracted extensive research interest for ceria-based catalysts due to its unique oxygen storage capacity, redox properties, abundant oxygen vacancies, broad-spectrum VOC degradation capability, excellent thermal stability, and tunable morphology [8,9,10]. For instance, CeO_2_ derived from an MOF-808 template (CeO_2_-MOF) [11] exhibits a significantly lower surface oxygen reduction temperature (480 °C) compared to conventional CeO_2_ (508 °C), along with a 20% increase in oxygen vacancy concentration, directly enhancing toluene oxidation efficiency. Secondly, their highly tunable structures and pore sizes make them widely applicable. The pore size range of MOFs (0.5–3 nm) bridges the gap between microporous zeolites and mesoporous silica, enabling selective catalysis of small molecules via a molecular sieving effect while suppressing carbon deposition from intermediates [12]. Finally, the unique structure of MOFs endows their derivatives with stable chemical properties and rich functionality; some MOFs remain stable under high temperatures (>400 °C) or humid conditions, and their catalytic performance can be enhanced through metal doping or post-synthetic modification. Wang [13] demonstrated that using mesoporous CeO_2_ derived from a Ce-BDC as a support for Pt nanoparticles resulted in richer surface defect sites, a 50% enhancement in toluene adsorption capacity, and a reduction in the reaction activation energy to 45 kJ/mol.

Noble metals play a pivotal role in the catalytic oxidation of VOCs. Noble metals (Pt, Pd, Au) serve as the preferred active components for VOCs oxidation catalysts due to their unique d-orbital electron configurations and high catalytic activity. Peng [14] reported that Pt/CeO_2_-r (rod-like) achieved complete toluene conversion (T90) at merely 150 °C, significantly lower than that of pure CeO_2_ (>300 °C) under identical conditions, indicating that Pt nanoparticles (NPs) activate oxygen species on the CeO_2_ surface via Pt-O-Ce bond formation, thereby reducing the reaction activation energy. Salaev M.A. et al. [15] demonstrated that loading pre-synthesized, size-controlled Pt colloidal solutions onto supports significantly enhanced Pt dispersion, consequently lowering both T10 (160 °C) and T90 (210 °C) for toluene oxidation, highlighting that tailoring the loading method can effectively modulate the state of noble metals and the metal–support interaction.

In summary, utilizing MOFs as precursors for catalyst preparation and modification represents a highly promising synthesis strategy, owing to advantages such as controllable morphology, high specific surface area, and abundant active sites. Furthermore, noble metal loading is currently an essential approach for substantially enhancing catalyst performance and enabling practical engineering applications. Therefore, investigating the methodology and sequence of loading on MOF-based precursors holds significant research importance, particularly in the field of thermal catalysis. This work focuses on CeO_2_-BTC to elucidate how different Pt loading methods, specifically impregnation, reverse impregnation, and reduction–co-assembly, lead to distinctions in actual Pt loading, effective chemical valence distribution, dispersion degree, cluster size, oxygen vacancy concentration, and Ce valence states. The origins of the high catalytic activity were elucidated through comprehensive characterization using XRD, Raman, TEM, XPS, H_2_-TPR, and DRIFTS. This work provides an experimental foundation and contributes strategic insights for developing noble metal-loaded oxide catalysts derived from metal–organic framework precursors.

## 2. Results and Discussion

**Figure 1:**
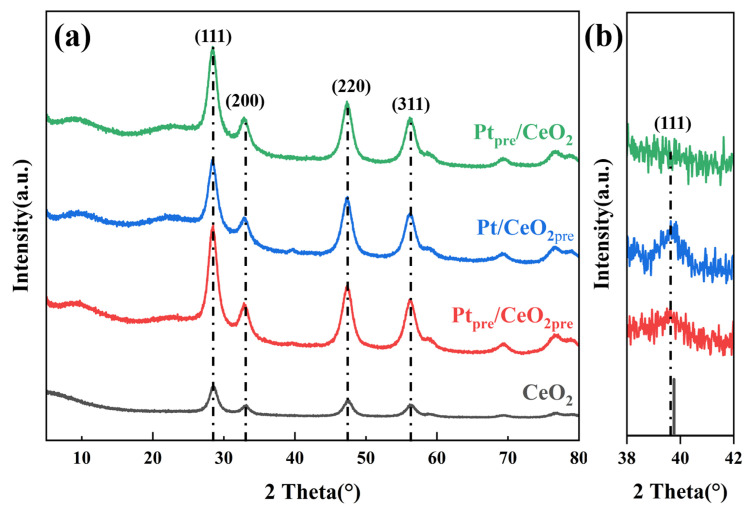
(**a**) XRD patterns of Pt_pre_/CeO_2_, Pt/CeO_2pre_, Pt_pre_/CeO_2pre_ and CeO_2_; (**b**) local magnified view of (**a**).

Figure 1 displays the XRD patterns of IM-prepared Pt_pre_/CeO_2_, RIM-prepared Pt/CeO_2pre_, and the RCM-prepared Pt_pre_/CeO_2pre_ catalysts synthesized with different loading sequences, and the CeO_2_ catalyst without noble metal loading. It is evident that the characteristic peaks of the catalysts with varying loading sequences all exhibit the typical cubic fluorite crystal phase of CeO_2_, where the peaks at 28.55, 33.07, 47.83, 56.34, 59.04, 69.4, 76.73, and 79.08° correspond to the (111), (200), (220), (311), (222), (400), (311), and (420) crystal planes of CeO_2_, respectively [15,16].

Upon magnifying the 2θ range of 39–41° for each catalyst, a characteristic peak is observed at 39.8°. It is identified as corresponding to PDF#00-023-1306 for Pt (111) crystal plane. Regarding the potential presence of trace amounts of Pt with other valence states, further analytical techniques are required for clarification. A distinct characteristic diffraction peak of Pt is observed for Pt/CeO_2pre_, indicating that a portion of the Pt exists as nanoparticles via this loading method. In contrast, this peak becomes less pronounced for Pt_pre_/CeO_2pre_, suggesting that Pt not only forms nanoparticles but also establishes chemical interactions with the support or exists in a highly dispersed state. The nearly undetectable Pt peak for Pt_pre_/CeO_2_ suggests that an even smaller fraction of Pt exists as nanoparticles. Considering the total Pt loading is as low as 1 wt%, the absolute amount of Pt in nanoparticulate form is too low to be detected by XRD. Furthermore, the Pt peaks of the three catalysts exhibit varying degrees of left or right shift, indicating alterations in lattice parameters. This indirectly suggests a change in electron attraction after Pt loading onto the CeO_2_ support, likely due to electron transfer between Pt and Ce [17], demonstrating the presence of metal–support interaction in the synthesized catalysts. Meanwhile, a quantitative comparison of the full width at half maximum (FWHM) of the (111) peak reveals a clear trend: the Pt-loaded catalysts exhibit progressively broader peaks than the pristine CeO_2_ support, following the order CeO_2_ < Pt/CeO_2pre_ < Pt_pre_/CeO_2_ < Pt_pre_/CeO_2pre_. This indicates that the Pt loading sequence does modify the crystallite size of CeO_2_, with the RCM method showing the most pronounced effect, likely due to stronger Pt–CeO_2_ interactions that inhibit CeO_2_ grain growth [18,19]. The reduced crystallite size may further influence the surface defect chemistry and, consequently, the catalytic performance.

**Figure 2:**
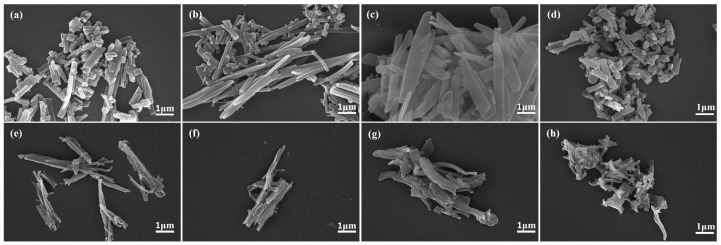
SEM of catalysts prepared by various loading methods: (**a**) Ce-BTC, (**b**) Pt_pre_/Ce-BTC, (**c**) Pt/Ce-BTC_pre_, (**d**) Pt_pre_/Ce-BTC_pre_, (**e**) CeO_2_, (**f**) Pt_pre_/CeO_2_, (**g**) Pt/CeO_2pre_, (**h**) Pt_pre_/CeO_2pre_.

Figure 2 shows the SEM images of the MOF precursors of catalysts and corresponding catalysts prepared by different loading methods, as well as the resulting catalysts after calcination. Figure 2a–c, and d correspond to the SEM images of the precursors Ce-BTC, Pt_pre_/Ce-BTC, Pt/Ce-BTC_pre_, and Pt_pre_/Ce-BTC_pre_, respectively. It can be observed that the as-synthesized Ce-BTC exhibits a nanorod-like morphology with non-uniform lengths and diameters ranging from tens to hundreds of nanometers. These nanorods aggregate to form bundled wood-like structures, which is consistent with previously reported morphologies [20]. Compared with Ce-BTC, the introduction of elemental Pt via different preparation sequences influences the precursors morphology to varying degrees. The Pt/Ce-BTC_pre_ sample exhibits preferential growth along a specific direction, resulting in flake-like structures resembling “Popsicle sticks”. For the Pt_pre_/Ce-BTC, some nanorods became elongated. In the image Pt_pre_/Ce-BTC_pre_, the nanorods of some crystals are shortened. The different Pt loading sequences and methods affect crystallization and morphology of the precursors; these morphological changes are likely attributable to the Pt loading inhibiting or promoting crystal growth along specific directions or influencing crystal nucleation under these loading conditions.

Figure 2e–g, and h present the SEM images of the CeO_2_, Pt_pre_/CeO_2_, Pt/CeO_2pre_ and Pt_pre_/CeO_2pre_ catalysts prepared by different loading methods, respectively. Compared with Figure 2a–d, Figure 2e–h reveal that the catalysts undergo bending deformation and surface roughening due to the removal of organic components during calcination, yet they largely retain the morphology of their MOF precursors. These differences in morphology and crystallization are certain to influence physicochemical properties such as surface area and Pt active site distribution, consequently leading to variations in catalytic activity.

**Figure 3:**
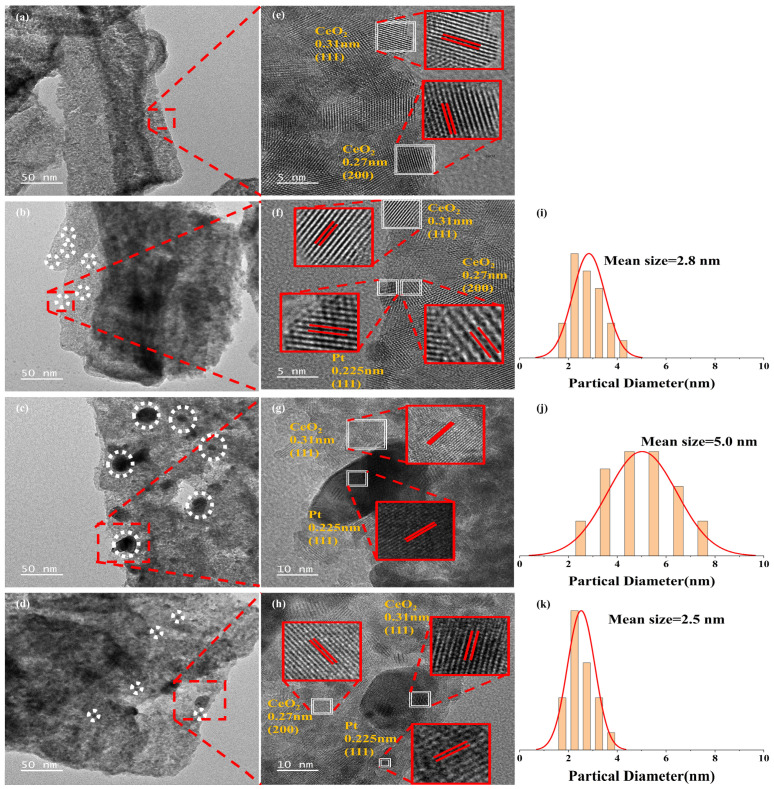
TEM images of catalysts prepared by various loading methods: (**a**) CeO_2_, (**b**) Pt_pre_/CeO_2_, (**c**) Pt/CeO_2pre_, (**d**) Pt_pre_/CeO_2_; (**e**–**h**) high-resolution images of (**a**–**d**); (**i**–**k**) particle size distribution of (**b**–**d**).

Figure 3a–d present TEM images of CeO_2_, Pt_pre_/CeO_2_, Pt/CeO_2pre_, and Pt_pre_/CeO_2pre_, with their corresponding high-magnification images shown in Figure 3e–h, respectively. Lattice fringes of the four catalysts can be clearly observed in high- resolution images. In Figure 3e, distinct lattice fringe spacings of 0.27 and 0.31 nm are visible, corresponding to the (200) and (111) crystal planes of CeO_2_, respectively. According to the literature [21], the (111) plane is the most stable crystal facet in CeO_2_, possessing a relatively low surface energy. Furthermore, due to its high density of oxygen vacancies and favorable catalytic activity [11], it is generally considered the most advantageous facet for thermal catalysis. The presence of Pt particles is evident in Figure 3b–d. Notably, the Pt particles in the Pt/CeO_2pre_ catalyst (Figure 3c) exhibit significant aggregation. In contrast, the Pt particles in the Pt_pre_/CeO_2_ (Figure 3b) and Pt_pre_/CeO_2pre_ (Figure 3d) catalysts are relatively well-dispersed and smaller in size. Lattice fringes with a spacing d of approximately 0.225 nm, corresponding to the Pt (111) plane, can be observed in Figure 3f–h. This confirms the successful loading of Pt onto the support surfaces, with exposure of the (111) plane, which exhibits high activity in thermal catalytic reactions [22,23].

Furthermore, multiple high-resolution TEM images were selected, and a large number of particles were measured for size statistics, resulting in the distribution plots shown in Figure 3i (Pt_pre_/CeO_2_), Figure 3j (Pt/CeO_2pre_), and Figure 3k (Pt_pre_/CeO_2pre_). The average particle sizes for Pt_pre_/CeO_2_, Pt/CeO_2pre_, and Pt_pre_/CeO_2pre_ are approximately 2.8, 5.0, and 2.5 nm, respectively. The corresponding Pt dispersion degree, calculated by using Equation (1) [24], is 40.16%, 22.49%, and 44.98%. The different loading methods significantly influence the Pt particle size and dispersion state. Compared to the 22.5% dispersion on Pt/CeO_2pre_ achieved via the impregnation method, both the reduction–co-assembly and reverse impregnation methods yielded better Pt dispersion. Numerically, the reduction–co-assembly method appears slightly superior. The agglomeration and dispersion state of Pt, along with its valence state, inherently influence the physicochemical state of the catalysts, consequently collectively affecting the catalytic oxidation activity of the material towards toluene.

Assuming Pt nanoparticles exhibit a spherical or hemispherical shape, the dispersion of Pt nanoparticles can be estimated using the following equation:

*D*_Pt_ = 6*M*_Pt_×100%/*ρdA*_Pt_N_A_, (1)

where *M*_Pt_ is the molar mass of Pt (195.08 g·mol^−1^), *ρ* is the density of Pt (21.45 g·cm^−3^), *d* is the average particle diameter observed from TEM images, *A*_Pt_ is the surface area of a Pt atom (8.06 × 10^−20^ m^2^·atom^−1^), and N_A_ is Avogadro’s constant (6.02 × 10^23^ mol^−1^). It is worth noting that Equation (1) herein is established under the assumptions that all particles are perfectly circular and that agglomeration effects are ignored. Therefore, the resulting calculations are likely to yield an overestimation, which may overstate the actual dispersion of Pt on the support surface.

**Figure 4:**
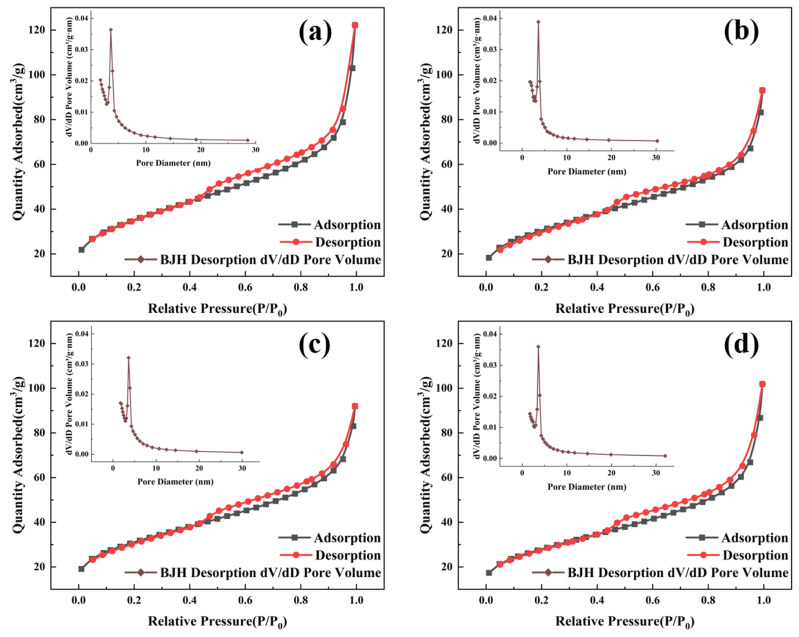
N_2_ adsorption–desorption isotherms of catalysts prepared by various loading methods: (**a**) CeO_2_, (**b**) Pt_pre_/CeO_2_, (**c**) Pt/CeO_2pre_, (**d**) Pt_pre_/CeO_2pre_.

Figure 4 represents the N_2_ adsorption–desorption isotherms and the corresponding pore size distribution curves for CeO_2_, Pt_pre_/CeO_2_, Pt/CeO_2pre_, and Pt_pre_/CeO_2pre_. Analysis of the N_2_ adsorption–desorption isotherms reveals that both the pure CeO_2_ and the catalysts prepared by different methods exhibit distinct Type IV isotherms, with H3-type hysteresis loops observed in the relative pressure range of 0.4–0.8. The H3-type hysteresis loops and the pore size distribution curves indicate the presence of a mesoporous structure in all four catalysts. These pores primarily originate from the collapse of the original MOF pores during calcination and the voids left by the removal of organic components from the MOF precursor. Although the specific surface area of typical Ce-MOFs is around 200 m^2^/g [19], which decreases after calcination for the pure CeO_2_ (122.3 m^2^/g), it is inferred that a significant number of pores and a relatively high specific surface area are still maintained since the CeO_2_ catalyst morphology is preserved post-calcination; specific data are provided in Table 1.

Table 1 presents the specific surface areas, average pore sizes, and pore volumes of the four catalysts. As shown in the table, all Pt supported catalysts maintain relatively high specific surface areas of approximately 100 m^2^/g. However, the values reveal that the specific surface areas of the Pt-supported catalysts prepared by different loading methods are all reduced to varying degrees compared to the unloaded CeO_2_. This can be attributed to two main factors: firstly, the different Pt loading methods affect the morphology of the catalyst precursors themselves; secondly, the particle size and dispersion degree of Pt may also influence the specific surface area and porosity of the catalysts.

**Figure 5:**
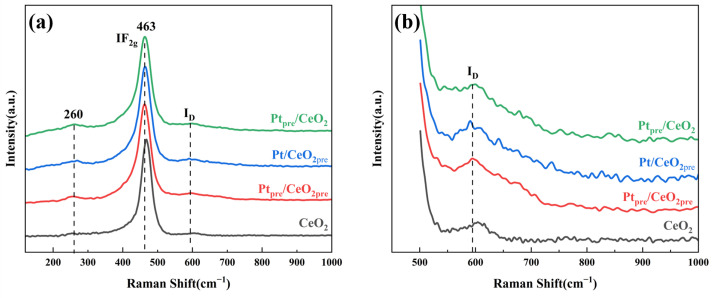
Raman spectra of catalysts prepared by various loading methods: (**a**) full-range spectrum; (**b**) Magnified spectrum.

Figure 5 shows the Raman spectra of CeO_2_ and the catalysts prepared by different loading methods, along with the detailed spectral profiles in the 500–1000 cm^−1^ range. A distinct characteristic peak is observed in the range of 400–600 cm^−1^, specifically at 463 cm^−1^, which corresponds to the symmetric stretching vibration mode (F_2g_) of the CeO_2_ fluorite lattice [25]. Upon magnification of the spectra, a peak located at approximately 591 cm^−1^ is visible in the 500–1000 cm^−1^ region. This peak is attributed to a defect-induced vibration mode associated with oxygen vacancies (D band), often linked to the presence of Ce^3+^ ions. The intensity ratio (I_D_/IF_2g_) serves as an indicator for estimating the relative concentration of oxygen vacancies in the catalysts. Consequently, the two characteristic peaks were integrated separately to obtain the I_D_/IF_2g_ ratios for the different catalysts.

Table 1 also lists the specific values of the I_D_/IF_2g_ ratios. The I_D_/IF_2g_ ratios for the catalysts follow the descending order: Pt_pre_/CeO_2pre_ > Pt/CeO_2pre_ > Pt_pre_/CeO_2_ > CeO_2_. This indicates that Pt_pre_/CeO_2pre_ possesses the highest relative concentration of oxygen vacancies among the series. Reported studies suggest that the catalytic activity of CeO_2_ is closely related to the symmetric stretching and asymmetric vibration intensities of the Ce-O bond [19,26]. Compared with pure CeO_2_, the catalysts prepared by the three different loading methods all exhibit a red shift in the F2g mode and a significant increase in the I_D_/IF_2g_ ratio, demonstrating that Pt loading effectively introduces oxygen vacancies, breaks the symmetry of Ce-O bond, and induces lattice distortion. The data confirm that Pt_pre_/CeO_2pre_ has the highest oxygen vacancy concentration. These results will be correlated with XPS measurements to further elucidate the oxygen vacancy concentrations in the different catalysts.

**Figure 6:**
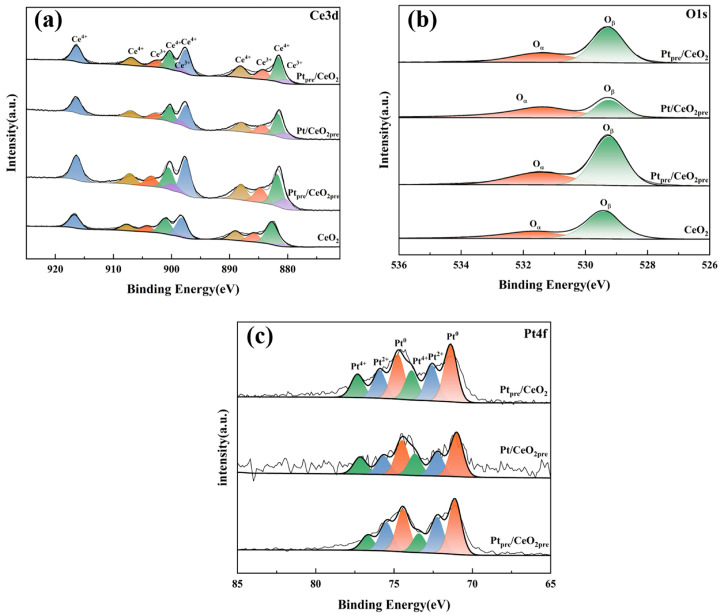
(**a**) Ce3d, (**b**) O1s, (**c**) Pt4f spectrum of catalysts prepared by different loading methods.

Figure 6a shows the Ce 3d XPS spectrum of CeO_2_ and the catalysts prepared by different loading methods. Peak deconvolution reveals 10 characteristic peaks. Taking the Pt_pre_/CeO_2pre_ catalyst as an example, the characteristic peaks are labeled as v_0_ (880.7 eV), v (882.7 eV), v′ (885.8 eV), v″ (888.6 eV), v‴ (898.3 eV), u_0_ (898.8 eV), u (901.5 eV), u′ (905.5 eV), u″ (907.3 eV), and u‴ (916.7 eV). The v peaks correspond to the Ce 3d_3/2_ spin–orbit component, while the u peaks correspond to the Ce 3d_5/2_ component. The peaks labelled v_0_, v′ and u_0_, u′ are assigned to Ce^3+^, whereas those labelled v, v″, v‴ and u, u″, u‴ are attributed to Ce^4+^, confirming the coexistence of both Ce^3+^ and Ce^4+^ species on all four catalysts. The quantitative results obtained from peak fitting and integration are listed in Table 2. It can be seen that the Ce^3+^/(Ce^3+^ + Ce^4+^) ratio is highest for the Pt_pre_/CeO_2pre_ catalyst at 34.9%, followed by Pt/CeO_2pre_ (24.4%), Pt_pre_/CeO_2_ (21.7%), and CeO_2_ (18.5%). This indicates that the reduction–co-assembly method, compared to the other two, promotes electron transfer (Pt → CeO_2_) via strong metal–support interactions (SMSIs), favoring a higher Ce^3+^ concentration. Given the concomitant relationship between Ce^3+^ and oxygen vacancies, a higher Ce^3+^ ratio often implies a greater abundance of oxygen vacancies in the material [27], which can enhance the catalytic oxidation of toluene. These findings are consistent with the Raman spectroscopy results.

To further identify the types of oxygen species, the chemical states of surface oxygen were examined. Figure 6b presents the O1s spectra of CeO_2_ and the catalysts prepared by different loading methods. All four samples exhibit two distinct characteristic peaks: the peak with a binding energy between 529 and 530 eV is assigned to surface lattice oxygen (O_β_) [28], while the peak in the 531–532 eV range corresponds to surface-adsorbed oxygen (O_α_) [29]. According to Table 2, the Ce^3+^ content of the three materials, Pt_pre_/CeO_2_, Pt/CeO_2pre_, and Pt_pre_/CeO_2pre_, increases in that order, with the Pt_pre_/CeO_2pre_ catalyst exhibiting the highest Ce^3+^ content. This is largely attributed to the strong metal–support interaction (SMSI) [30]. A higher Ce^3+^ content tends to keep Pt in the metallic state (Pt^0^) or promote the reduction of Pt^4+^/Pt^2+^ to Pt^0^. Consequently, the latter two catalysts show relatively high Pt^0^ content, with Pt_pre_/CeO_2pre_ displaying the highest Pt^0^ content. However, an excessively high Ce^3+^ concentration leads to an overly high surface electron density, creating a strongly reducing environment (excessively negative chemical potential) that suppresses the chemisorption of oxidative adsorbed oxygen species (e.g., O_2_^−^, O^−^). Although the concentration of oxygen vacancies is the highest, the surface “reduction potential” becomes the dominant factor, resulting in a net decrease in adsorbed oxygen content. Therefore, the observed trend of initially increasing and then decreasing surface-adsorbed oxygen content among the three materials is a typical characteristic of strong SMSI effects. Additionally, the three different Pt introduction methods also affect the surface Pt content of the catalysts, with the IM method (Pt_pre_/CeO_2_) yielding the highest surface Pt loading. The actual Pt loading, surface Pt loading, Pt particle size, distribution, valence state, and their interaction with Ce collectively determine the subsequent catalytic oxidation performance for toluene. This interaction promotes the dynamic generation of oxygen vacancies on the CeO_2_ surface and enhances oxygen mobility [14,17,30,31].

Figure 6c shows the Pt4f spectra of the catalysts prepared by different loading methods. Six characteristic peaks were observed in the Pt 4f region for all samples. Taking Pt_pre_/CeO_2_ as an example, its spectrum can be deconvoluted into three spin–orbit doublets. The doublet located at 71.11 eV (v_1_) and 74.44 eV (u_1_), with a spin–orbit splitting of 3.33 eV, corresponds to the 4f_7/2_ and 4f_5/2_ orbitals of metallic Pt^0^. The doublet at 72.25 eV (v_2_) and 75.49 eV (u_2_), with a splitting of 3.24 eV, is assigned to oxidized Pt^2+^ species. The doublet at 73.41 eV (v_3_) and 76.69 eV (u_3_), with a splitting of 3.28 eV, is attributed to oxidized Pt^4+^ species [32,33].

As shown in Table 2, the Pt^0^/Pt ratios for the three samples Pt_pre_/CeO_2_, Pt/CeO_2pre_, and Pt_pre_/CeO_2pre_ are 46.5%, 49.2%, and 49.7%, while the Pt^2+^/Pt ratios are 29.3%, 26.6%, and 32.8%. It is reported in the literature [4,15,33] that Pt^0^ serves as the active site for toluene adsorption, lowering the dissociation energy barrier of the C-H bond. The role of Pt^2+^ is to adsorb and dissociate O_2_ molecules via surface oxygen vacancies, generating active oxygen species that participate in the deep oxidation of toluene. Among all the samples, Pt_pre_/CeO_2pre_ exhibits the highest relative contents of Pt^0^ and Pt^2+^. Additionally, Table 2 reveals that the actual Pt loading efficiencies for all three methods are above 90%, and notably, the RMC method achieves a loading efficiency exceeding 95%, which might be associated with the better dispersion of Pt species. However, the noble metals are not homogenously distributed in the spatial volume of the catalysts under normal conditions; instead, they are preferentially localized on the external surface, especially for catalysts where Pt is loaded onto pre-synthesized supports exemplified by the IM-prepared Pt_pre_/CeO_2_. This surface-enrichment effect explains the higher-surface Pt loading (up to 6%, Table 2) observed for this catalyst. Since the surface physicochemical properties critically govern the catalytic performance, we also measured and present the surface Pt contents for all three materials in Table 2.

**Figure 7:**
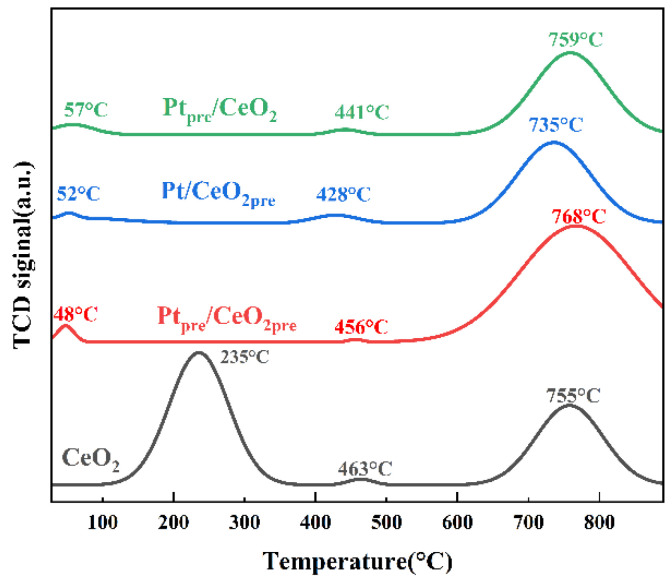
H_2_-TPR of catalysts prepared by various loading methods.

Figure 7 presents the H_2_-TPR profiles of the catalysts prepared by different loading methods. For CeO_2_, three characteristic reduction peaks are observed at 235, 463, and 755 °C, corresponding to the reduction in surface-adsorbed oxygen, surface lattice oxygen, and bulk lattice oxygen of CeO_2_, respectively [11,34,35]. After Pt loading, the characteristic peak in the 200–300 °C range disappears for the Pt_pre_/CeO_2_, Pt/CeO_2pre_, and Pt_pre_/CeO_2pre_ catalysts, and is replaced by a new low-temperature reduction peak appearing around 50 °C.

According to literature reports [27,36], the surface oxygen (O-Ce) reduction peak of CeO_2_ could be replaced by a reduction peak corresponding to the Pt-O-Ce interface, which is attributed to the reduction in oxidized Pt^2+^ and Pt^4+^ to metallic Pt^0^ at the interface, a state more favorable for catalytic activity. Among them, the Pt_pre_/CeO_2pre_ catalyst prepared by reduction–co-assembly exhibits the lowest temperature for this peak (48 °C) and the largest peak area. This indicates that the Pt_pre_/CeO_2pre_ catalyst possesses relatively strong redox capability at lower temperatures. Furthermore, in the high-temperature reduction region (600–900 °C), the order of catalyst peak areas is Pt_pre_/CeO_2pre_ > CeO_2_ > Pt_pre_/CeO_2_ > Pt/CeO_2pre_. This shows that the Pt_pre_/CeO_2pre_ catalyst has the highest bulk oxygen concentration, indicating that the introduction of Pt via the reduction–co-assembly method generates more bulk oxygen and improves the oxygen storage capacity. In the Mars–van Krevelen (MVK) reaction mechanism, lattice oxygen participates in the catalytic oxidation reaction and is a core factor for catalytic activity [37,38,39]. Therefore, the Pt_pre_/CeO_2pre_ catalyst prepared by the reduction–co-assembly method, with its lower reduction temperature and higher lattice oxygen concentration, is expected to exhibit excellent catalytic activity. Additionally, the initiation temperatures for the high temperature peaks of Pt/CeO_2pre_ are lower, suggesting that it also holds an advantage in redox capability within certain temperature ranges.

**Figure 8:**
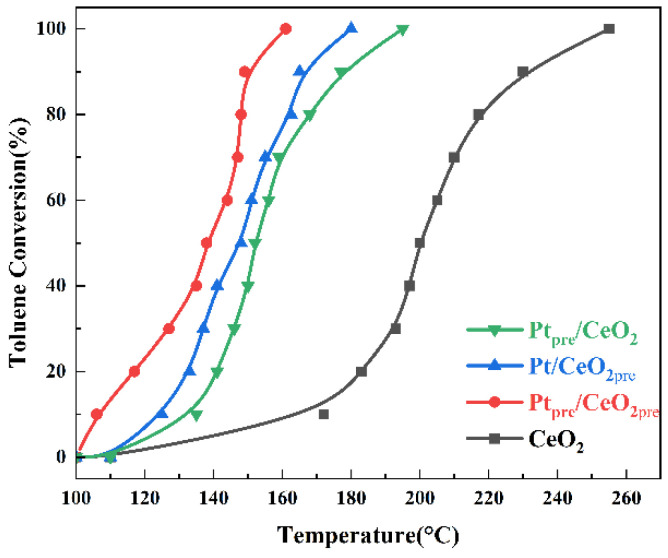
Performance of catalysts prepared by different loading methods. (Toluene concentration = 1000 ppm, GHSV = 40,000 mL·g^−1^·h^−1^).

To evaluate the toluene catalytic oxidation performance of different Pt loading methods, catalytic performance tests were conducted on CeO_2_ and the three Pt-loaded catalysts under conditions of a toluene concentration of 1000 ppm and a space velocity of 40,000 mL·g^−1^·h^−1^. The results are shown in Figure 8 and Table 3.

It can be observed that all three Pt loading methods significantly enhance catalytic activity, albeit to varying degrees. Among them, the Pt_pre_/CeO_2pre_ catalyst exhibits the highest catalytic activity. Compared to the T_10_ of CeO_2_ (177 °C), the T_10_ values for the Pt_pre_/CeO_2_, Pt/CeO_2pre_, and Pt_pre_/CeO_2pre_ catalysts are 135, 125 and 106 °C, respectively, representing decreases of 42, 52 and 71 °C. This indicates that loading Pt significantly improves the ignition temperature (T_10_) of the catalyst. The improvement is most pronounced for the Pt_pre_/CeO_2pre_ catalyst. When the toluene conversion reaches 50%, the temperature differences (ΔT_50_) for the Pt_pre_/CeO_2_, Pt/CeO_2pre_ and Pt_pre_/CeO_2pre_ catalysts compared to CeO_2_ are 48, 52 and 62 °C. It can be seen that during this stage, the toluene conversion temperature for Pt_pre_/CeO_2pre_ remains substantially lower. When the toluene conversion reached 90%, the temperatures (T_90_) for the Pt_pre_/CeO_2_, Pt/CeO_2pre_ and Pt_pre_/CeO_2pre_ catalysts are lower than that of CeO_2_ by 53, 65 and 81 °C. At complete toluene conversion, the temperatures for the Pt_pre_/CeO_2_, Pt/CeO_2pre_ and Pt_pre_/CeO_2pre_ catalysts were lower than that of CeO_2_ by 60, 75 °C and 94 °C. Thus, it is evident that Pt loading significantly enhances catalyst activity throughout the 50% to 100% toluene conversion range, particularly for the Pt_pre_/CeO_2pre_ catalyst.

Table 4 summarizes the catalytic oxidation performance (T_90_) for toluene over recently reported catalyst systems with similar compositions [14,24,40,41,42,43,44,45]. The T_90_ value of our as-prepared Pt_pre_/CeO_2pre_ catalyst remains below 150 °C, indicating that it exhibits relatively excellent catalytic activity for toluene oxidation. In terms of noble metal loading, our catalyst possesses a moderate level. It should be noted that our prepared catalyst does not afford the highest activity or the lowest Pt loading among the compared systems; nevertheless, this work provides valuable insights into the sequence of noble metal loading on oxide catalysts derived from metal–organic framework (MOF) precursors, as well as the resulting metal dispersion, particle size, and valence state distribution.

**Figure 9:**
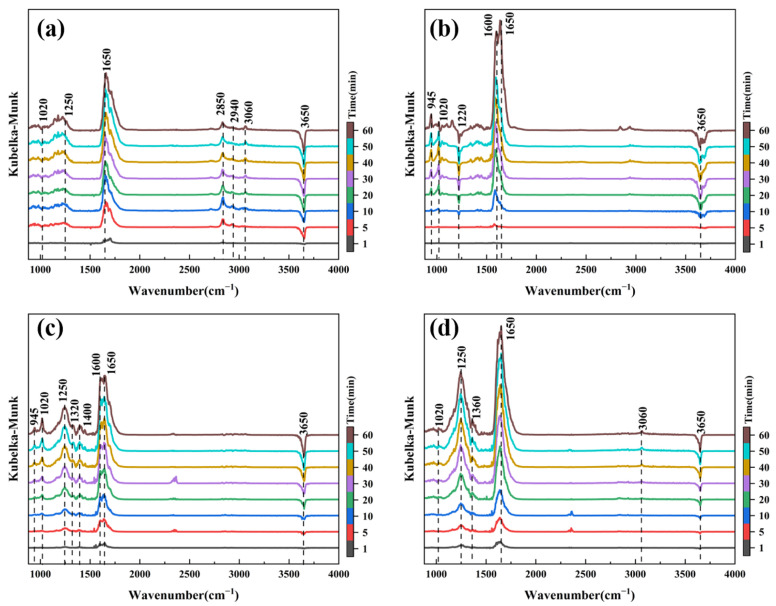
In situ DRIFTS of toluene adsorption on catalysts without oxygen on (**a**) CeO_2_, (**b**) Pt_pre_/CeO_2_, (**c**) Pt/CeO_2pre_, (**d**) Pt_pre_/CeO_2pre_.

Figure 9 shows the in situ diffuse reflectance infrared Fourier transform spectrum (DRIFTS) on CeO_2_ and the various catalysts during the catalytic oxidation of toluene in an oxygen-free atmosphere. According to literature reports [38,46], the vibrational band at 1020 cm^−1^ is assigned to the C–O stretching vibration of benzyl alcohol, while the bands at 1600 and 3060 cm^−1^ correspond to the skeletal vibration of the aromatic ring and the C–H stretching vibration of the benzene ring, respectively. The bands at 1360 and 2940 cm^−1^ are attributed to the symmetric deformation vibration of methyl (–CH_3_) groups or the C–H deformation vibration in benzoic acid/benzaldehyde [39,46]. The bands at 945 cm^−1^ (C–H out-of-plane bending of benzaldehyde), 1220 and 1250 cm^−1^ (C–O stretching of benzoic acid), 1320 cm^−1^ (O–H bending of benzaldehyde), 1650 cm^−1^ (C=O stretching of benzaldehyde or C–O vibration of benzoate species), and 2850 cm^−1^ (C–H stretching of benzaldehyde) are all assigned to key intermediate products, namely benzaldehyde or benzoic acid species [39,47,48]. The band at 3650 cm^−1^ corresponds to the O–H stretching vibration of surface hydroxyl groups [49].

Upon removal of surface-adsorbed oxygen species during pretreatment, the adsorption and conversion of toluene rely entirely on the lattice oxygen species of the catalyst surface. Figure 9 presents in situ DRIFT spectra of toluene adsorption over four catalysts after removal of surface-adsorbed oxygen, recorded from 1 min to 60 min. For CeO_2_ (Figure 9a), characteristic vibrations of benzaldehyde and benzoate at 1650 cm^−1^ and the aromatic ring skeletal vibration at 1600 cm^−1^ appear within 1 min of toluene introduction, indicating rapid oxidation of toluene by surface lattice oxygen to oxygenated intermediates. After 5 min, peaks corresponding to benzyl alcohol (1020 cm^−1^), benzoic acid (1250 cm^−1^), and methyl/aromatic C–H (2940/3060 cm^−1^) gradually intensify. Meanwhile, a negative band at 3650 cm^−1^ suggests consumption of hydroxyl groups by toluene oxidation [50]. At 50 min, methyl-related peaks (2850 and 2940 cm^−1^) slightly decrease. This may be attributed to the further conversion of some intermediates or the formation of carbon deposits, and at this stage, the lattice oxygen may also be insufficient to support further reaction.

In contrast, Pt_pre_/CeO_2_ (Figure 9b) shows slower initial activation: a weak peak at 1600 cm^−1^ emerges at 5 min, and key intermediate bands at 945, 1020, and 1650 cm^−1^, along with negative bands at 1220 and 3650 cm^−1^, appear only at 10 min. Nevertheless, the intensity of the 1650 and 1600 cm^−1^ peaks increases significantly and continuously up to 60 min, with other peaks stabilizing after 40 min. This indicates that although the initial reaction rate of Pt_pre_/CeO_2_ is lower than that of CeO_2_, Pt promotes CeO_2_ lattice oxygen mobility, effectively replenishing surface active oxygen and enabling sustained reaction without carbon deposition. For Pt/CeO_2pre_ (Figure 9c), positive peaks at 1250, 1600, and 1650 cm^−1^ and a negative peak at 3650 cm^−1^ are detected within 1 min. At 5 min, additional peaks at 945, 1020, 1320, and 1400 cm^−1^ appear, with all peak intensity continuing to rise until the reaction ends. This suggests rapid toluene adsorption and activation, as well as good lattice oxygen mobility that effectively promotes the reaction.

Notably, Pt_pre_/CeO_2pre_ (Figure 9d) exhibits superior performance. Characteristic vibration peaks at 1250, 1360, 1600, and 1650 cm^−1^, along with a negative band at 3650 cm^−1^, appear immediately upon toluene introduction. The intensity of all intermediate peaks increases continuously throughout the reaction without any attenuation. Moreover, compared with Figure 9c, the number of intermediate vibration bands is significantly smaller, and some peaks (e.g., 945 cm^−1^) are absent, indicating an extremely fast reaction rate over this sample. These results demonstrate that, in the absence of gas-phase oxygen replenishment, surface lattice oxygen of CeO_2_ serves as the primary active oxygen species for toluene oxidation. The introduction of Pt significantly enhances lattice oxygen mobility and promotes the adsorption and activation of toluene molecules.

**Figure 10:**
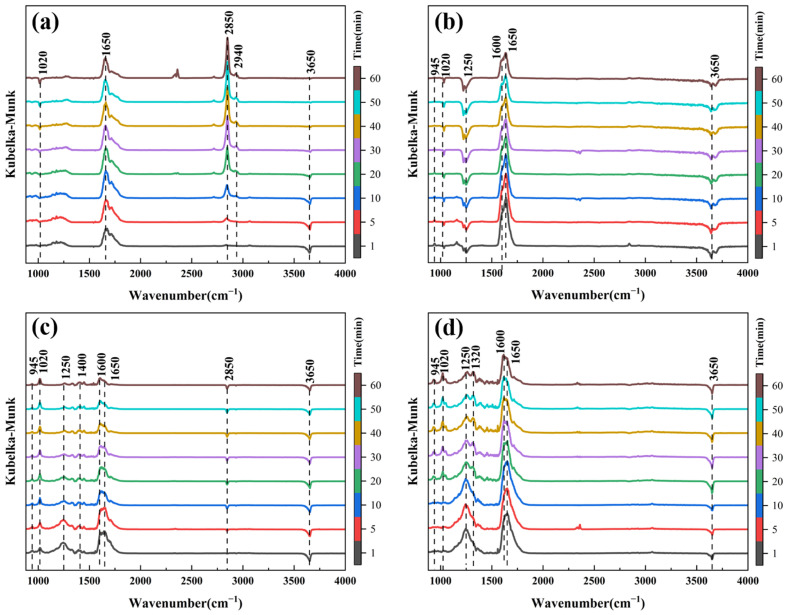
In situ DRIFTS of toluene adsorption on catalysts with oxygen on (**a**) CeO_2_, (**b**) Pt_pre_/CeO_2_, (**c**) Pt/CeO_2pre_, (**d**) Pt_pre_/CeO_2pre_.

Figure 10 presents the in situ DRIFTS of toluene adsorption over the catalysts for 1–60 min with the surface-adsorbed oxygen species retained. For CeO_2_ (Figure 10a), strong vibrational peaks attributed to benzoic acid (1220, 1250, and 1700 cm^−1^) and the aromatic ring skeleton (1600 cm^−1^) appear within 1 min, along with a distinct negative peak at 3650 cm^−1^. After 5 min, peaks at 2850 and 2940 cm^−1^ emerge, while a weak negative peak appears at 1020 cm^−1^. Subsequently, the peak at 2850 cm^−1^ continuously intensifies, and the negative peak at 3650 cm^−1^ disappears at 40 min, indicating that the consumption and regeneration of surface hydroxyl groups reach equilibrium, yet the accumulation of abundant benzoic acid intermediates on the material surface hinders further reaction.

Compared with Figure 10a, Figure 10b shows that within the first 1 min of reaction, characteristic vibrational peaks at 945, 1600, 1650, and 2850 cm^−1^ appear simultaneously, along with negative peaks at 1020, 1250, and 3650 cm^−1^. The peak at 2850 cm^−1^ disappears after 5 min, and the intensity of the 1650 and 1600 cm^−1^ peaks gradually decreases as the reaction proceeds. This phenomenon suggests that, over the Pt_pre_/CeO_2_ sample, although Pt species facilitate the adsorption and activation of toluene, the loss of surface active sites occurs during the reaction, which may be related to carbon deposition on the material surface. Owing to the loss of active sites, the concentration of residual intermediates on the material surface decreases, leading to weakened characteristic peaks. Pt/CeO_2pre_ (Figure 10c) also exhibits a decreasing trend in intermediate peaks. However, starting from 5 min, the intensity of all peaks except that at 1020 cm^−1^ declines rapidly, and a negative peak appears at 2850 cm^−1^. After 40 min, all intermediate peaks become relatively weak, indicating that carbon deposits cover a large number of active sites, resulting in catalyst deactivation.

For Pt_pre_/CeO_2pre_ (Figure 10d), a large number of characteristic intermediate peaks (945, 1020, 1250, 1320, 1600, 1650 cm^−1^) along with a negative peak at 3650 cm^−1^ are observed at the initial stage of the reaction. As the reaction proceeds, the peaks at 945 and 1020 cm^−1^ continuously intensify, whereas the peaks at 1250, 1600, and 1650 cm^−1^ gradually weaken, with changes stabilizing after 20 min. This phenomenon indicates that, although some loss of active sites also occurs over the Pt_pre_/CeO_2pre_ catalyst, the loss is minor, allowing the accumulation of intermediates such as benzyl alcohol (1020 cm^−1^) and benzaldehyde (945 cm^−1^) to still be observed. Meanwhile, the generation and consumption of benzoic acid species (1250, 1600, 1650 cm^−1^) eventually reach a moderate balance, suggesting that although carbon deposition exists, it does not impair the catalytic performance of this catalyst. The above in situ DRIFTS results demonstrate that Pt loading promotes the adsorption and oxidation of toluene, with Pt_pre_/CeO_2pre_ exhibiting the best catalytic oxidation performance for toluene, followed by Pt/CeO_2pre_.

In summary, different Pt loading methods affect the distribution, particle size, chemical state of Pt, and the metal–support interaction on the catalyst surface. The three catalysts (IM-prepared Pt_pre_/CeO_2_, RIM-prepared Pt/CeO_2pre_, and RCM-prepared Pt_pre_/CeO_2pre_) all achieved actual loading efficiencies of nearly 90%, with the RCM method showing a slightly higher efficiency (4–5%). Regarding the surface Pt loading, the IM method yielded the highest value. The IM-prepared Pt_pre_/CeO_2_ and RCM-prepared Pt_pre_/CeO_2pre_ catalysts exhibited smaller Pt particle sizes and more uniform distribution (Table 2). In contrast, the RIM-prepared Pt/CeO_2pre_ and RCM-prepared Pt_pre_/CeO_2pre_ catalysts possessed higher contents of active Pt species and Ce^3+^. Furthermore, in terms of surface oxygen activation and toluene adsorption, the three materials followed the order: Pt_pre_/CeO_2pre_ > Pt/CeO_2pre_ > Pt_pre_/CeO_2_. For the Pt_pre_/CeO_2pre_ catalyst, which exhibits the best catalytic oxidation performance for toluene, the introduction of Pt species promotes the activation of oxygen and facilitates the adsorption and activation of toluene. Moreover, Based on comprehensive characterization including TEM, Raman, XPS, N_2_ adsorption–desorption isotherms, H_2_-TPR, and O_2_-TPD, we infer that the superior activity of Pt_pre_/CeO_2pre_ originates from multiple synergistic factors: its ultralow reduction temperature, high relative concentration of oxygen vacancies, high Ce^3+^ fraction, high proportions of Pt^0^ and Pt^2+^ species, homogeneous dispersion of active Pt, and a moderate lattice oxygen concentration. These properties collectively enhance oxygen activation and toluene adsorption/activation, thereby accounting for the outstanding catalytic performance of this material.

The sequence of Pt introduction in the three methods affects Pt dispersion, active Pt valence states, and surface Pt content, which in turn influence the oxygen vacancy concentration and Ce^3+^ content on CeO_2,_ resulting in different redox properties and toluene adsorption performances. Moreover, Pt introduction at different stages alters the morphology and crystallinity of the CeO_2_ support, as well as its intrinsic activity for toluene oxidation. Variations in Pt dispersion, as well as the crystallite size and morphology of CeO_2_, also affect the specific surface area. Collectively, these factors synergistically govern the catalytic activity.

Meanwhile, based on the above results, it can be found that when no gas-phase oxygen species are replenished during the reaction, the reaction can proceed depending on the lattice oxygen on the catalysts’ surface. When gas-phase oxygen species are replenished, the Pt species can effectively promote the activation of gas-phase oxygen, enabling the toluene oxidation reaction to proceed rapidly and continuously. Furthermore, a comparative analysis of the characteristic peaks obtained under oxygen-free and oxygen-containing environments reveals that the main pathway for toluene degradation over the catalyst synthesized by the chemical reduction loading method is: toluene → benzaldehyde → benzoic acid → ring-opening products (carboxylic acids) → CO_2_ + H_2_O.

## 3. Materials and Methods

### 3.1. Chemicals and Materials

Cerium nitrate hexahydrate (Ce(NO_3_)_3_·6H_2_O, 99.9%), chloroplatinic acid hexahydrate (H_2_PtCl_6_·6H_2_O, 99.9%) and Trimesic acid (H_3_BTC, 98%) were purchased from Macklin (Shanghai, China). Polyvinylpyrrolidone (PVP, K30) and sodium borohydride for synthesis (NaBH_4_, 98%) were obtained from Tianjin Zhiyuan Reagent Co., Ltd. (Tianjin, China). Sodium citrate tribasic dihydrate (C_6_H_5_Na_3_O_7_·2H_2_O, 99%) was purchased also from Macklin (Shanghai, China). Deionized water (DI water) and anhydrous ethanol (C_2_H_5_OH) used in the experiment were supplied by Tianjin Fuyu Chemical Co., Ltd. (Tianjin, China).

### 3.2. Synthesis of Catalysts

#### 3.2.1. Synthesis of CeO_2_ Nanorod Catalyst

Ce(NO_3_)_3_·6H_2_O (4.34 g, 10 mmol) was dissolved in 30 mL of DI water to form Solution A. Solution B, in addition, was obtained by dissolving the H_3_BTC (2.1 g, 10 mmol) in 30 mL of anhydrous ethanol. Then, Solution B was transferred into a water bath and heated to 60 °C. Solution A was added to Solution B dropwise and kept for 3 h. The resulting product, Ce-BTC, was then collected by centrifugation, washing and drying. The prepared Ce-BTC was calcined in air 320 °C for 2 h at a heating rate of 5 °C min^−1^ to obtain the CeO_2_ nanorod catalyst.

#### 3.2.2. Synthesis of Pt_pre_/CeO_2_ Catalyst

Samples of 1.47 g C_6_H_5_Na_3_O_7_·2H_2_O and 0.0096 g PVP (K30) were dissolved in 30 mL of DI water. In addition, 4.665 mL chloroplatinic acid hexahydrate (3.75 mg/mL) was added into the mixed solution. The precursor solution of Pt nanoparticles was thus formed through stirring the above mixture at room temperature for 30 min. Then, the precursor solution of Pt nanoparticles was poured into 30 mL of DI water containing Ce-BTC (10 mmol) to obtain 1 wt.% Pt-to-CeO_2_ final ratio in the catalyst. The mixed solution was thoroughly stirred for 30 min and placed in a water bath with ice water. Slightly excessive sodium borohydride (1.5 mmol) was added into the mixture and stirred for 30 min. So, the precursor product, Pt_pre_/Ce-BTC, was prepared after the above reaction product was centrifuged, washed and dried. Finally, Pt_pre_/CeO_2_ was collected while the precursor product was calcined in air 320 °C for 2 h at a heating rate of 5 °C·min^−1^.

#### 3.2.3. Synthesis of Pt/CeO_2pre_ Catalyst

1 wt.% Pt nanoparticles were synthesized by the sodium borohydride reduction method. Typically, 1.47 g C_6_H_5_Na_3_O7·2H_2_O and 0.0096 g PVP (K30) were dissolved in 30 mL of DI water. A 4.665 mL chloroplatinic acid hexahydrate (3.75 mg/mL) sample was then added into the mixed solution. After 30 min stirring, the mixture was transferred to a water bath with ice water. A solution containing Pt nanoparticles was precipitated by adding slightly excessive sodium borohydride (1.5 mmol) and reacting for 30 min. Afterwards, the above solution was mixed with Solution A (10 mmol) and stirred at room temperature for 30 min. The mixture was dropwise added to Solution B (10 mmol) in the water bath and kept at 60 °C. After 3 h of constant temperature stirring, Pt/Ce-BTC_pre_, the precursor product, was obtained by centrifugation, washing and drying. Pt/CeO_2pre_, the final product, was collected by calcining in air 320 °C for 2 h at a heating rate of 5 °C·min^−1^.

#### 3.2.4. Synthesis of Pt_pre_/CeO_2pre_ Catalyst

**Figure 11:**
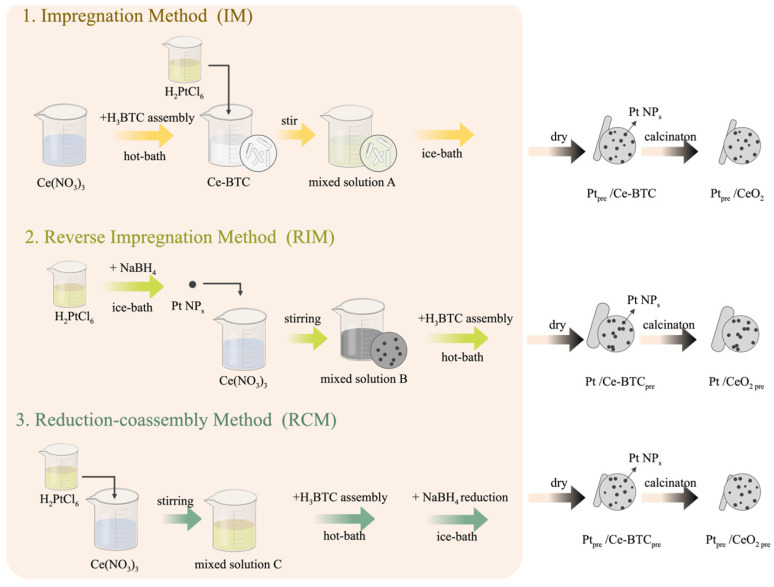
Schematic diagram of different Pt loading sequences using MOF as the precursor.

The required amount of precursor solution of Pt nanoparticles containing 1.47 g C_6_H_5_Na_3_O_7_·2H_2_O, 0.0096 g PVP (K30), and 4.665 mL chloroplatinic acid hexahydrate (3.75 mg/mL) to obtain a 1 wt.% Pt-to-CeO_2_ final ratio in the catalyst derived from Pt_pre_/Ce-BTC_pre_ was dissolved in 30 mL of DI water. Then, the solution was poured into Solution A and stirred for 30 min. The mixed solution was dropwise-added into Solution B, which was put into a water bath, heated to 60 °C, and stirred for 3 h. The reaction product was transferred into a water bath with ice water, cooled to room temperature, and was then slowly added and stirred for 30 min with slightly excessive sodium borohydride (1.5 mmol) to prepare the mixture of precursor. The precursor, Pt_pre_/Ce-BTC_pre_, was collected by centrifugation, washing and drying. The required final product, Pt_pre_/CeO_2pre_, was obtained after the precursor product was calcined in air 320 °C for 2 h at a heating rate of 5 °C·min^−1^. The synthesis process of the three catalysts above is shown in Figure 11.

### 3.3. Characterization

The morphology images of precursor MOF and their oxides were taken by a scanning electron microscope (SEM, FEI Sirion 200, Eindhoven, The Netherlands) at 20 kV, as well as a transmission electron microscopy (TEM, Talos F200s, Thermo Fisher, Waltham, MA, USA). An Accelerated Surface Area and Porosimetry System (Micromeritics ASAP 2460, Norcross, GA, USA) was used to analyze the Brunauer–Emmert–Teller (BET) surface area and pore structure. The mass fraction of Pt in the oxides was obtained by an inductively coupled plasma optical emission spectrometer (ICP-OES, Agilent 5110, Santa Clara, CA, USA). X-ray diffraction (XRD, Rigaku SmartLab SE, Osaka, Japan) and X-ray photoelectron spectroscopy (XPS, Thermo Scientific K-Alpha, Waltham, MA, USA) were used to characterize the crystalline structure and elemental valence of samples. Lattice defects and changes in oxygen vacancy concentration due to the addition of precious metals were obtained with XPS and Raman spectrum (Raman, Horiba LabRAM HR Evolution, Kyoto, Japan). The adsorption test of materials (H_2_-TPR) was performed on a TP5080D automatic chemical adsorption instrument (Xianquan, Tianjin, China). Nicolet 6700 FTIR spectrometer (Perkin Elmer, Shelton, Connecticut, USA) was used to characterize in situ diffuse reflectance infrared Fourier transform spectroscopy (DRIFTS) and then demonstrate catalytic intermediates of toluene oxidation.

### 3.4. Catalytic Activity Test

The catalytic oxidation process of toluene was operated on a fixed quartz reactor. A 150 mg sample (40–60 mesh) and 300 mg quartz sand (40–60 mesh) were mixed thoroughly and put into the quartz tube. The concentrations of toluene was 1000 ppm, the feed was 7.92 mL·min^−1^, and the weight hourly space velocity (GHSV) was 40,000 mL·g^−1^·h^−1^. The flame ionization detector (FID) was equipped on a gas chromatography to analyze online the end gas. The catalytic activity was characterized by the conversion of toluene. Normally, T10, T50 and T90, which were defined as the temperature of the percentage of toluene consumption concentration to initial toluene concentration at 10%, 50% and 90%, were used to illustrate the conversion of toluene.

## 4. Conclusions

In this work, the chemical reduction loading method was employed, using sodium borohydride to reduce chloroplatinic acid, followed by calcination of the Ce-BTC carrier to form the metal oxide catalyst. Three catalysts were prepared with different sequences of Pt source introduction: the impregnation method yielded Pt_pre_/CeO_2_, the inverse impregnation method yielded Pt/CeO_2pre_, and the reduction–co-assembly method yielded Pt_pre_/CeO_2pre_. The chemical reduction loading method enhances the stability of the catalyst’s crystal structure and alleviates the agglomeration of catalyst grains. Moreover, among the three catalysts, Pt_pre_/CeO_2pre_ exhibits the smallest Pt particle size, as determined by XRD, SEM, TEM, and N_2_ adsorption–desorption isotherm tests. Pt_pre_/CeO_2pre_ shows the highest oxygen vacancy concentration and best low-temperature reducibility (Raman, H_2_-TPR), as well as the highest proportions of Ce^3+^, O_α_, and Pt^0^ species (XPS), all of which are favorable for toluene oxidation. The Pt introduction sequence has a significant impact on catalytic activity, with Pt_pre_/CeO_2pre_ displaying the best performance (T_90_ = 149 °C).

## Figures and Tables

**Table 1:** Structural property data of catalysts prepared by various loading methods.

Catalysts	Specific Surface Area m^2^/g	Pore Diameter nm	Pore Volume mL/g	Pt Distribution %	I_D_/IF_2g_ ^1^ %
CeO_2_	122.3	4.82	0.12	-	2.44
Pt_pre_/CeO_2_	97.2	5.62	0.11	40.2	4.18
Pt/CeO_2pre_	107.5	4.86	0.10	22.5	5.68
Pt_pre_/CeO_2pre_	106.9	5.94	0.13	45.0	11.5

^1^ Calculated based on Raman results from Figure 5b.

**Table 2:** XPS data analysis results of catalysts prepared by different loading methods.

Catalysts	Actual Pt Loading Amount ^1^ wt%	Ce^3+^/(Ce^3+^ + Ce^4+^) %	O_α_/(O_α_ + O_β_) %	Pt^0^/Pt^2+^/Pt^4+^ %	Surface Loading Amount of Pt ^2^ wt%
CeO_2_	-	18.5	35.7	-	
Pt_pre_/CeO_2_	0.90	21.7	33.3	46.5/29.3/24.2	6.0
Pt/CeO_2pre_	0.91	24.4	53.5	49.2/26.6/24.2	2.6
Pt_pre_/CeO_2pre_	0.95	34.9	30.0	49.7/32.8/17.5	3.7

^1^ determined by ICP, ^2^ determined by XPS.

**Table 3:** Catalytic oxidation performance of toluene over catalysts prepared by different loading methods.

Samples	T_10_ (°C)	T_50_ (°C)	T_90_ (°C)	T_100_ (°C)
CeO_2_	177	200	230	255
Pt_pre_/CeO_2_	135	152	177	195
Pt/CeO_2pre_	125	148	165	180
Pt_pre_/CeO_2pre_	106	138	149	161

**Table 4:** Comparison on toluene oxidation over various Pt-loaded CeO_2_ composite catalysts.

Catalysts	T_90_ (°C)	Pt Loading Amount wt%	Ref
Pt/CeO_2_-r	150	0.19	[14]
Pt/CeO_2_-1.8	143	0.25	[24]
Single-atom Pt-CeO_2_/Co_3_O_4_	169	0.06	[40]
Dielectric barrier discharge plasma modified Pt/CeO_2_-PT6	220	0.77	[41]
Pt/CeO_2_-N	152	0.43	[42]
1Pt-MS-CeO_2_ hollow Nanospheres	171	1.00	[43]
Pt/CeO_2_-HA	155	0.71	[44]
Pt/CeO_2_-0.5	170	1.00	[45]

## Data Availability

Data available on request due to restrictions.
